# Comparison of decarboxylation rates of acidic cannabinoids between secretory cavity contents and air-dried inflorescence extracts in *Cannabis sativa* cv. ‘Cherry Wine’

**DOI:** 10.1038/s41598-024-66420-3

**Published:** 2024-07-16

**Authors:** Eun-Soo Kim, Sang-Hyuck Park, Chad A. Kinney, Kenneth J. Olejar, Ingrid Carolina Corredor-Perilla

**Affiliations:** 1 Institute of Cannabis Research, Colorado State University-Pueblo, Pueblo, CO 81001 USA; 2 Department of Chemistry, Colorado State University-Pueblo, Pueblo, CO 81001 USA

**Keywords:** *Cannabis*, Cannabinoids, Decarboxylation, Glandular trichome, Secretory cavity, Inflorescence, Biochemistry, Chemical biology

## Abstract

Studies with secretory cavity contents and air-dried inflorescence extracts of the CBD-rich hemp strain, *Cannabis sativa* cv. ‘Cherry Wine’, were conducted to compare the decarboxylation rates of acidic cannabinoids between two groups. The secretory cavity contents acquired from the capitate-stalked glandular trichomes by glass microcapillaries, and inflorescence samples air-dried for 15 days of storage in darkness at room temperature were analysed by high-pressure liquid chromatography. The ratio of acidic cannabinoids to the total cannabinoids was ranging from 0.5% to 2.4% lower in the air-dried inflorescence samples compared to the secretory cavity samples as follows. In the secretory cavity content, the percentage of acidic cannabinoids to the total cannabinoids was measured as 86.4% cannabidiolic acid (CBDA), 6.5% tetrahydrocannabinolic acid (THCA), 4.3% cannabichromenic acid (CBCA), 1.4% cannabigerolic acid (CBGA), and 0.6% cannabidivarinic acid (CBDVA), respectively. In the air-dried inflorescence, however, the acidic cannabinoids were detected with 84% CBDA, 4.8% THCA, 3.3% CBCA, 0.8% CBGA, and 0.3% Δ^9^-tetrahydrocannabivarinic acid (Δ^9^-THCVA), respectively. The ratio of cannabidiol (CBD) to cannabidiolic acid (CBDA) was close to 1:99 (w/w) in secretory cavity contents, however, it was roughly 1:20 (w/w) in the air-dried inflorescence. In addition, Δ^9^-tetrahydrocannabivarin (Δ^9^-THCV) and Δ^9^-tetrahydrocannabivarinic acid (Δ^9^-THCVA) were only detected in the air-dried inflorescence sample, and the ratio of Δ^9^-THCV to Δ^9^-THCVA was about 1:20 (w/w). Besides, cannabidivarinic acid (CBDVA) was only observed in the secretory cavity content.

## Introduction

Recently, 750 chemical compounds including 150 cannabinoids have been identified in *Cannabis* plants^1,2^. Cannabinoids are the active constituents of the *Cannabis* plant. Due to their potential, significant therapeutic and medicinal properties, cannabinoids have been addressed in various disease indications since ancient times^3–9^. More recently, the antiviral activities of cannabinoids against SARS-CoV-2, HIV, and COVID-19 have been demonstrated, and the demand for research on their medical effects has urgently emerged^10–14^.

The biosynthesis of cannabinoids has been elucidated over the past decades^15–17^. Cannabinoids exist in two forms: the prevalent carboxylated form, commonly referred to as acidic cannabinoids, and the decarboxylated form, neutral cannabinoids primarily derived from the transformation of acidic cannabinoids^18^. Further, more than 60 cannabinoids are formed by non-enzymatic modification reactions like decarboxylation, isomerization, and oxidation^19^.

The decarboxylation reaction of acidic cannabinoids occurs naturally in plants. The term ‘decarboxylation’ is a widespread organic chemistry reaction, which is the loss of carboxylic acid in the form of carbon dioxide^20^. Consequently, acidic cannabinoids are called ‘pre-cannabinoids’ by some researchers^18^. The converted cannabinoids such as Δ^9^-THC and CBD are pharmacologically more active in the human body than their acidic counterpart^21,22^. On the other hand, Rock *et al*.^23^ reported that THCA is 10 times more potent than Δ^9^-THC^24^, and CBDA may be 100-1000 times more potent than CBD in reducing nausea and vomiting in animal model^25^.

Currently, clinical interest in THCA and CBDA have been growing due to the lack of psychoactivity, however, their decarboxylation reaction remains a challenging issue. If acidic cannabinoid stability is solved with the stabilizing matrix, cannabis products of acidic cannabinoids will increase dramatically in the market.

In *Cannabis*, heating, drying, or combustion initiate the transformation reaction of cannabinoids, and these processes can alter the profile of cannabinoids^26^. For example, these phenomena often lead to the decarboxylation of acidic cannabinoids and the eventual formation of neutral cannabinoids such as cannabidiol (CBD), cannabigerol (CBG), and Δ^9^-tetrahydrocannabinol (Δ^9^-THC)^27–29^.

Cannabidiolic acid (CBDA) and tetrahydrocannabinolic acid (THCA) are the primary components of *Cannabis* inflorescence, along with minor components such as cannabichromenic acid (CBCA), cannabigerolic acid (CBGA), and cannabinolic acid (CBNA)^30^. While these compounds have been detected to a lesser extent in other parts of the plants, CBDA and THCA predominantly accumulate in the secretory cavities of capitate-stalked and sessile glandular trichomes^31,32^. In particular, the capitate-stalked glandular trichomes produce the highest concentrations of cannabinoids in the secretory cavities^33,34^.

Acidic cannabinoids including CBDA and THCA have been reported to induce apoptosis in both plant cells and insect cells, suggesting their cytotoxic properties and potential role as plant defence compounds^35^. THCA has been associated with hydrogen peroxide formation, potentially contributing to the self-defence mechanism of *Cannabis*^36^. Nonetheless, cannabinoids have been observed to cause mitochondrial permeability transition, DNA degradation, and cell necrosis in plants and cancer^37,38^.

Cannabinoids are deposited and stored in the extracellular cavity called a secretory cavity of glandular trichomes to prevent cellular damage, which has been suggested as the site of the final step in cannabinoid biosynthesis^39,40^. The cannabinoid biosynthesis process is initiated in the cytosol of disc cells and concludes with oxidocyclization in the apoplastic space, at or outside the plasma membrane^41,42^.

The plastids of disc cells are recognized as the primary site for acidic cannabinoid biosynthesis in glandular trichomes and subsequent accumulation in the secretory cavity^43,44^. However, the exact areas of cannabinoid conversion remain unclear.

In this study, the secretory cavity contents and the air-dried inflorescence extracts were examined to compare the cannabinoid profiles and to track the progress of acidic cannabinoid decarboxylation. Given that the conversion of acidic cannabinoids into neutral or transformed cannabinoids yields significantly different pharmacological effects, these findings will contribute crucial insights into the control of manufacturing processes for the pharmaceutical and industrial applications of cannabinoids.

## Results

**Figure 1:**
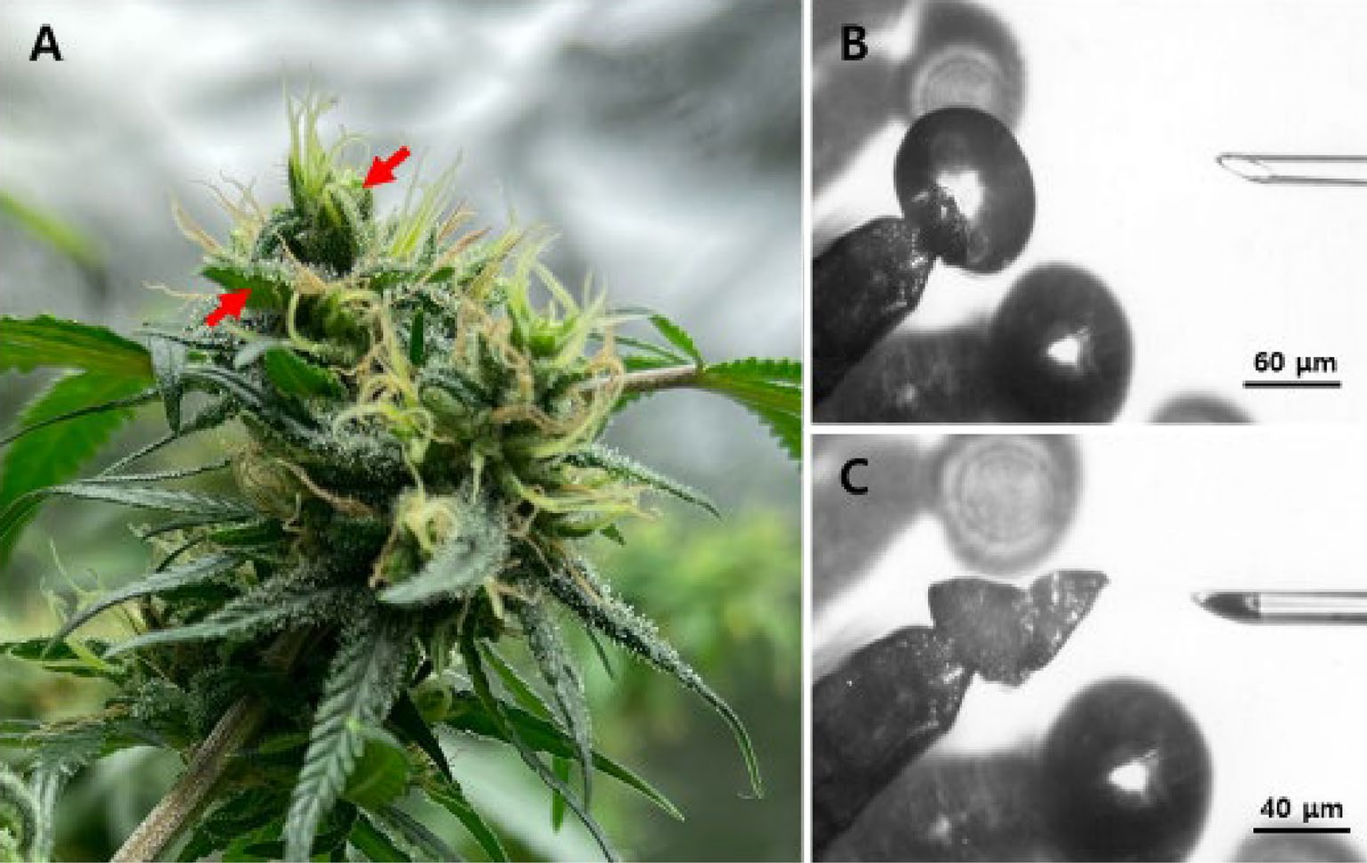
Female cannabis inflorescence and stalked glandular trichomes. (**A**) Cannabis inflorescence with a cluster of calyxes and bracts (arrows) containing numerous glandular trichomes. (**B**, **C**) Snapshots demonstrating the isolation of the secretory cavity contents by glass microcapillary. An intact glandular head and an approaching glass microcapillary are visible (**B**). Collapsed glandular head and collected secretory cavity contents in the tip of a glass microcapillary (**C**).

**Figure 2:**
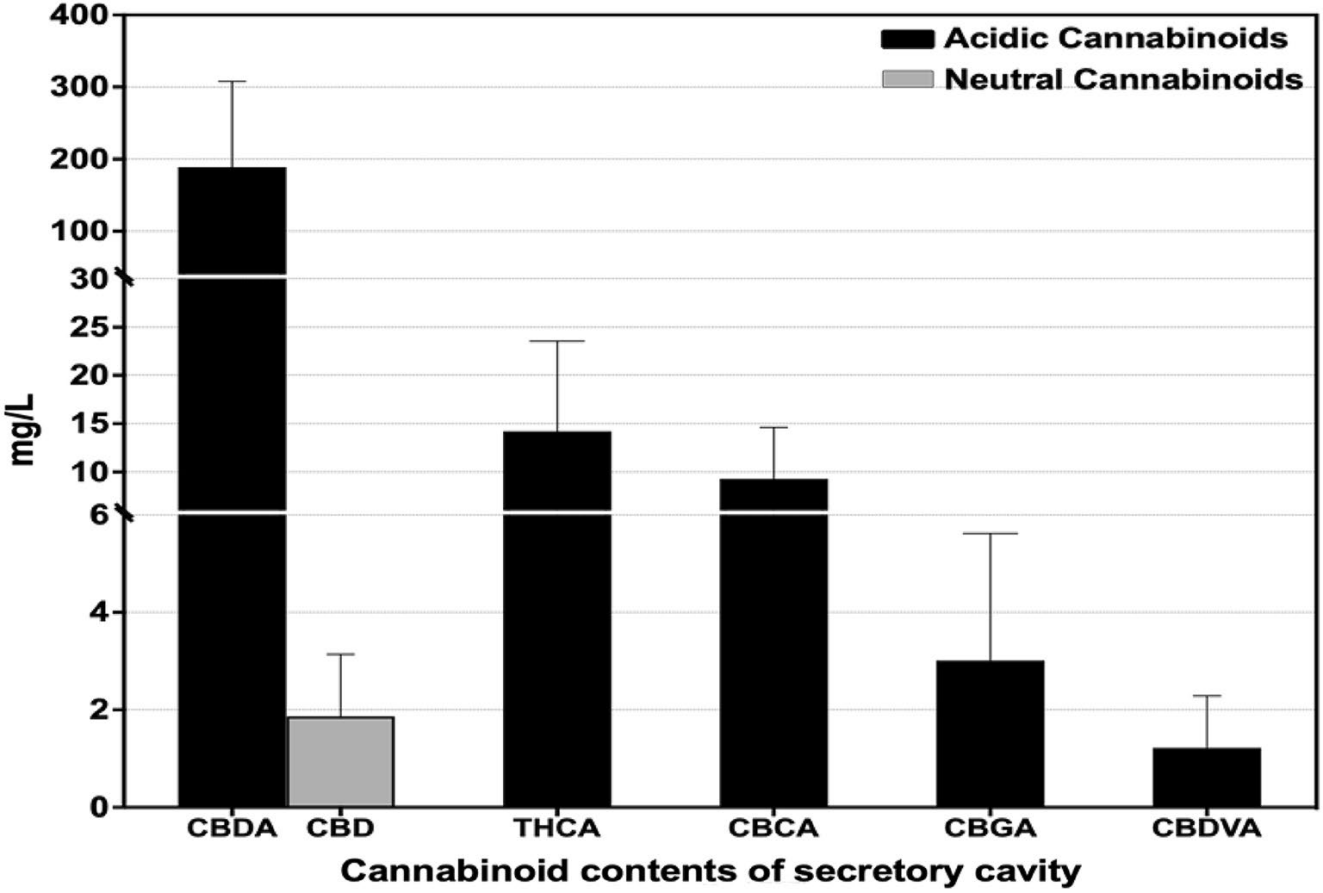
Cannabinoid profile of the secretory cavity contents acquired by glass microcapillaries.

The major and minor cannabinoids were qualitatively and quantitatively analysed by an external standard HPLC method. For the fresh sample, we employed glass microcapillaries to acquire the secretory cavity contents from capitate-stalked glandular trichomes in the pistillate calyx (Fig. 1). The secretory cavity contents exhibited detectable quantities of nine cannabinoids, with high levels of CBDA, THCA, CBCA, CBGA, and CBDVA. All acidic cannabinoids, except Δ^9^-THCVA, were present at their highest concentrations in the secretory cavity with the following composition (Fig. 2). The major cannabinoid content was determined as follows: CBDA (189.0 ± 68.8 mg/L), CBD (1.90 ± 0.7 mg/L), THCA (14.2 ± 5.4 mg/L), CBCA (9.3 ± 3.1 mg/L), CBGA (3.0 ± 1.5 mg/L), and CBDVA (1.2 ± 0.6 mg/L) were observed in the secretory cavity contents, respectively (Table 1).

**Table 1:** Cannabinoid profile detected in secretory cavity contents.

Cannabinoids contents (mg/L)				
Compounds	Minimum	Maximum	Mean	± SD*
CBDA	61.73	297.97	189	68.8
CBD	0.75	3.26	1.9	0.7
Δ^9^-THCA	4.63	23.3	14.2	5.4
CBCA	3.58	14.16	9.3	3.1
CBGA	ncd**	4.70	3.0	1.5
CBDVA	ncd**	1.96	1.2	0.6

*Mean ± standard error, n = 3 (40 glands per sample).

**ncd, no cannabinoids detected.

**Figure 3:**
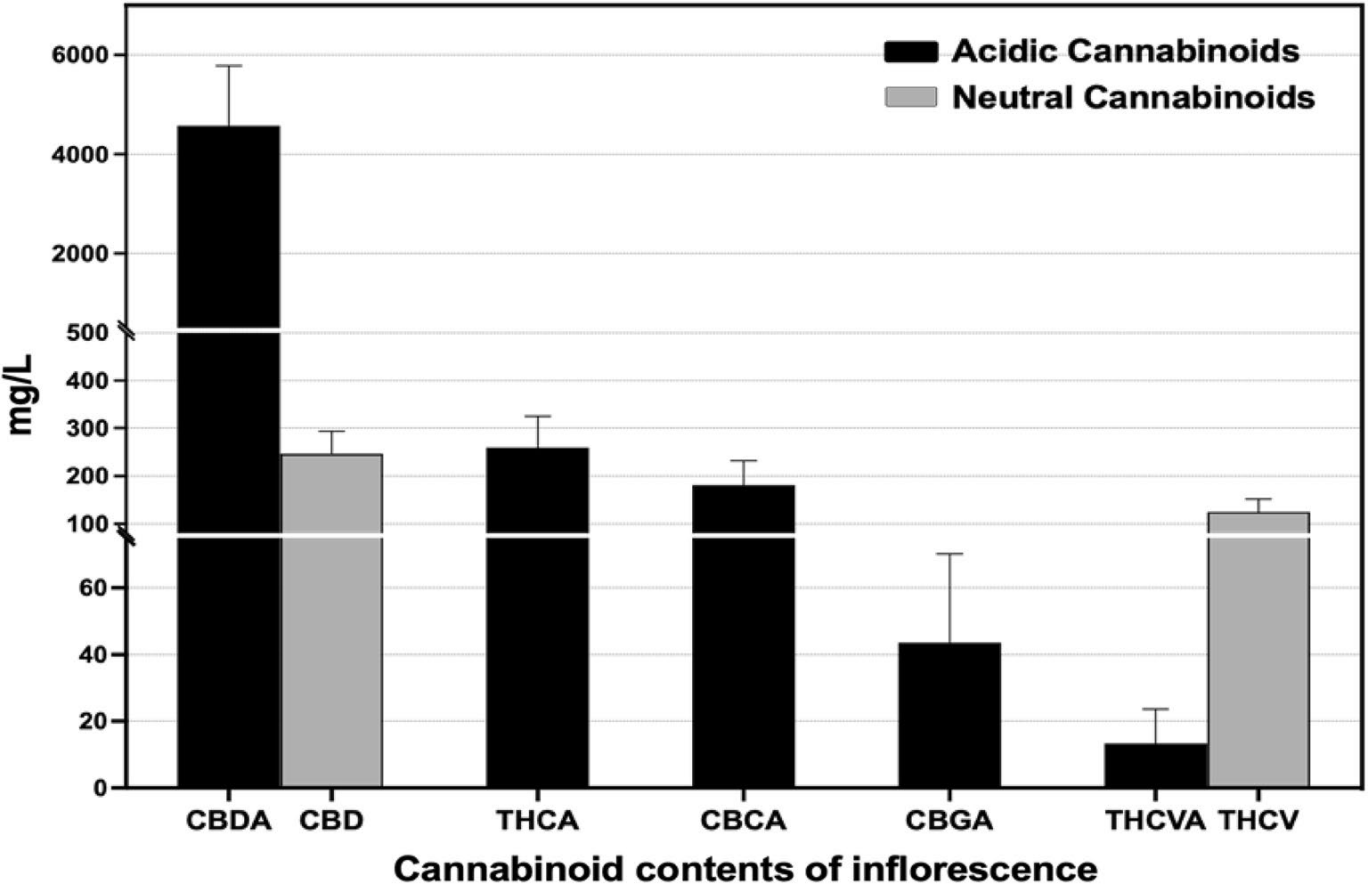
Cannabinoid profile of the air-dried inflorescence.

There are no qualitative differences in acidic cannabinoid profiles between the secretory cavity contents and the inflorescence samples excluding Δ^9^-THCVA and CBDVA (Fig. 3). The cannabinoid profile of the air-dried inflorescence samples exhibited substantially more decarboxylated cannabinoids compared to the secretory cavity contents as follows: CBDA (4575.0 ± 493.2 mg/L), CBD (246.3 ± 18.2 mg/L), THCA (259.2 ± 26.9 mg/L), CBCA (181.1 ± 20.9 mg/L), CBGA (43.6 ± 10.9 mg/L), Δ^9^-THCVA (13.4 ± 4.2 mg/L), and Δ^9^-THCV (125.0 ± 10.9 mg/L) in the inflorescence sample (Table 2). The dried inflorescence samples contained both Δ^9^-THCVA and Δ^9^-THCV, while the secretory cavity contents uniquely possessed CBDVA.

**Table 2:** Cannabinoid profile detected in air-dried inflorescence.

Cannabinoids contents (mg/L)				
Compounds	Minimum	Maximum	Mean	± SD*
CBDA	2621.8	5801.1	4575	493.2
CBD	190.7	305.7	246.3	18.2
Δ^9^-THCA	141.7	318.7	259.2	26.9
CBCA	96.7	226.7	181.1	20.9
CBGA	6.2	15.5	43.6	10.9
Δ^9^-THCVA	2.3	22.1	13.4	4.2
Δ^9^-THCV	73.9	145.2	125	10.9

*Mean ± standard error, n = 6.

**Figure 4:**
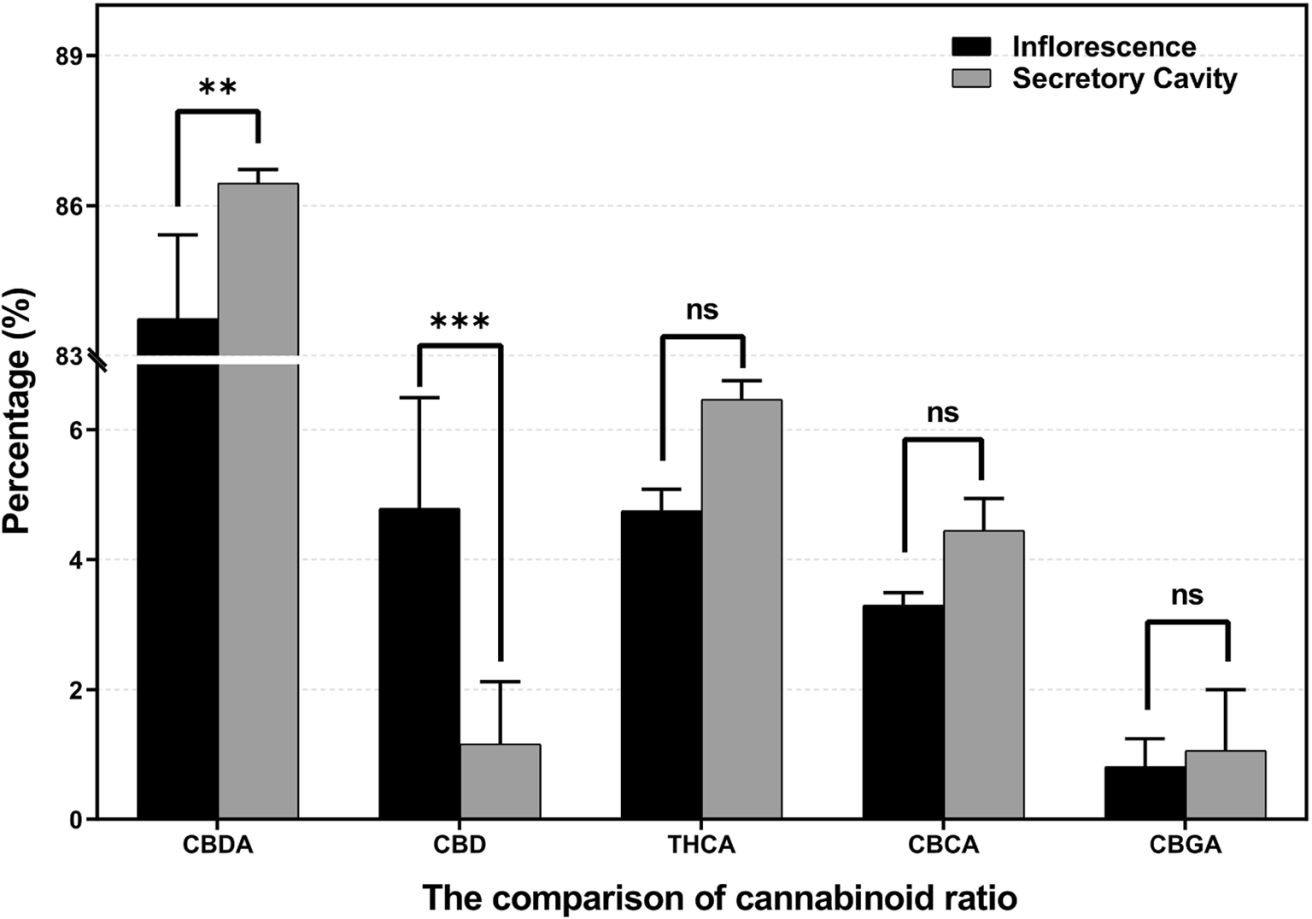
Comparison of the percentage of acidic and neutral cannabinoids of inflorescence extracts and secretory cavity contents. Data was analyzed using two-way ANOVA. **p < 0.01, ***p < 0.001, ns: not significant (*p* > 0.05).

The secretory cavity contents showed a higher proportion of CBDA (86.45 ± 0.28%) compared to the air-dried inflorescences extracts (83.74 ± 1.67%). However, the content of CBD in the dried inflorescences was much higher (4.79 ± 1.70%) compared to that of the secretory cavity samples (1.16 ± 0.97%) (Fig. 4).

Consequently, the secretory cavity contents showed relatively higher levels close to 99% of carboxylated cannabinoids in their profile compared to that of the air-dried inflorescence extracts, which is roughly 93%. In the air-dried inflorescence extracts, all acidic forms of cannabinoids have been reduced ranging from 0.5% to 2.4% to the corresponding neutral form of the cannabinoids compared to that of the secretory cavity contents (Tables 1 and 2).

In addition, the cannabinoids contained in the secretory cavity of a glandular trichome were analysed. Although the cannabinoid content might not be equal in quantity in all glandular trichomes of the plant, we measured the average amounts of cannabinoids of the individual glandular trichomes as follows: CBDA (234.0 ng/gland), CBD (5.0 ng/gland), THCA (35.0 ng/gland), CBCA (22.5 ng/gland), CBGA (7.5 ng/gland), and CBDVA (7.5 ng/gland) (Table 3). The other cannabinoids were either not detected or detected below the quantification concentrations in both samples.

**Table 3:** Average amounts of cannabinoids present in a glandular trichome.

Compounds	CBDA	CBD	Δ^9^-THCA	CBCA	CBGA	CBDVA
Amounts (ng)	234	5	35	22.5	1.5	1.5

## Discussion

Phytocannabinoids are produced by the *Cannabis* plant’s metabolism in the form of carboxylic acids. They are classified into three groups: acidic cannabinoids (carboxylated group, *e.g.*, CBDA, CBGA, CBCA, and THCA), neutral cannabinoids (decarboxylated group, *e.g.,* CBD, CBG, CBC, and Δ^9^-THC), and transformed cannabinoids (degraded group, *e.g.,* CBNA, CBN, Δ^8^-THC, CBL, and CBLA)^16^.

Both the glandular trichomes and inflorescence in *Cannabis* contain terpenes, flavonoids, and cannabinoids^31^. The major site of acidic cannabinoid synthesis and accumulation has been confirmed in glandular trichomes^33,43^. Also, the final process of acidic cannabinoid decarboxylation has been widely accepted as non-enzymatic reactions in nature^29^. Secretory substances synthesized from plastids are secreted into the cytosol and transported beneath a thin waxy membrane surrounding the disc cells of glandular trichomes. In these extracellular spaces, the secreted compounds are segregated from the disc cells, protecting them from both oxidative degradation and enzyme change^39,41^. We suspect that these spaces may be possible sites for the transformation or decarboxylation of cannabinoids, free from environmental factors such as oxygen, enzymes, and light.

### Transformation of acidic cannabinoids

During the storage of cannabis plants, the contents of its major cannabinoids vary depending on storage conditions such as light, darkness, dryness, and temperature^45^. Notably, the major psychoactive cannabinoid, Δ^9^-THC, is relatively unstable and may undergo changes when the biomass is stored long-term^46^. The impact of temperature on decarboxylation was initially demonstrated by comparing drying temperature. Drying at 37 °C compared to 60 °C resulted in higher concentration of acid cannabinoids^47^.

Taschwer and Schmid^46^ reported that storage temperatures at 100 °C and 150 °C led to a complete decarboxylation of THCA to Δ^9^-THC within short time while storage temperature at 50 °C led to slight changes of THCA concentration ranged from 12.2 to 11.7%. Thus, processing and storage temperature of cannabis products may be crucial factors in causing decarboxylation of acidic cannabinoids and degradation of neutral cannabinoids. As the optimum temperature for the decarboxylation of THCA to Δ^9^-THC, a research team proposed 150 °C to get a 70% yield^48^. Also, it was reported that the decarboxylation kinetics of CBDA to CBD provided an estimation for cannabinoid stability in hempseed product^49^.

Some researchers reported that the relative trichome concentration of Δ^9^-THC and CBD is 1–2% of that of their precursors THCA and CBDA, as determined by LCMS^34^. As shown in Fig. 4, the secretory cavity contents demonstrated higher proportion of CBDA (86%) compared to the air-dried inflorescences extracts (84%). Conversely, the content of CBD in air-dried inflorescences (4.5%) was observed higher than that of the secretory cavity samples (0.9%). Eventually, the ratio of CBD to CBDA was 1: 99 (w/w) in secretory cavity contents, but 1:20 (w/w) in the inflorescence sample.

Based on our results that the amount of CBD increases fivefold in 15 days of storage, we can predict the conversion rate of acidic cannabinoids and use these data to manage hemp products. Although the secretory cavity contents showed fewer kinds of cannabinoids, it contained more amounts of acidic cannabinoids as follows. As compared the cannabinoid profile of the air-dried inflorescence extracts with that of the secretory cavity contents, 2.4% of CBDA, 1.7% of THCA, 0.9% of CBCA, and 0.5% of CBGA was decreased, respectively.

In addition, no Δ^9^-THC, CBC, and CBG were detected in the secretory cavity contents and the inflorescence samples except for Δ^9^-THCV and Δ^9^-THCVA in the former sample, and CBDVA in the latter sample. In fibrous *Cannabis*, the ratio of CBD to CBDA may be a useful indicator of acidic cannabinoid decarboxylation in biomass samples, as CBDA typically predominates in the extract.

In fresh plants, all cannabinoids are theoretically present in the acidic form but are found together with the neutral form. Capitate-stalked trichomes most actively synthesize and produce acidic cannabinoids, with the concentration increasing during the female plant flowering period^29,33^. Our results reveal that just an air-drying procedure without lighting or heating can induce a chemical transformation reaction, converting a measurable fraction of acidic cannabinoids into neutral cannabinoids.

Depend on De Backer team’s data, a theoretical existence of 92% of THCA and 8% Δ^9^-THC has often been used as basis for the theoretical content calculation^50^. Therefore, we suggest the ratio of Δ^9^-THC/THCA or CBD/CBDA can be used as a marker for evaluation of quality and determination of the shelf-life in cannabis-based products.

### Degraded cannabinoids and their characteristics

Δ^9^-THCV is a homologue of Δ^9^-THC, rendering it a minor cannabinoid with non-psychoactive properties. Higher amounts of Δ^9^-THCV are characteristically present in fibrous strains of *Cannabis*^51^. The absence of Δ^9^-THCVA and Δ^9^-THCV in the secretory cavity contents suggests that these cannabinoids have not been transformed yet. However, the presence of Δ^9^-THCV and Δ^9^-THCVA in the inflorescence sample indicates that the drying process of the samples leads to the conversion of acidic cannabinoids to neutral or degraded cannabinoids simultaneously. Cannabidivarin (CBDV) is a structural analogue of CBD, featuring a shorter side chain^28^. Under acidic conditions, CBDV isomerizes into Δ^9^- THCV, and in vivo CBDV is the precursor of Δ^9^-THCV^52^.

Our result revealed high levels of Δ^9^-THCV, accounting for approximately 2.3% of total cannabinoids in the dried inflorescence sample. In this research, CBDV was not observed in either the secretory cavity or inflorescence samples, but a small amount of CBDVA was detected in the secretory cavity contents. This result indicates that the high concentration of CBDA in the secretory cavity might potentially influence the transformation of CBDA into CBDVA.

Microcapillary-based extraction facilitates a comprehensive analysis of both acidic and neutral cannabinoids, providing valuable insights into cannabinoid transformation such as decarboxylation. Consequently, selective sampling from secretory cavities enables the rapid cannabinoid analysis by eliminating the environmental and influencing factors during the extraction process. Overall, the utilization of microcapillaries exhibits significant potential for advancing cannabinoid research as well as finding practical applications in the industrial sector.

## Methods

### Plant materials

A CBD-rich strain, *Cannabis sativa* cv*.* ‘Cherry Wine’ (CW) seeds were purchased from High Grade Hemp Seeds (Pueblo, Colorado) and used in this study. This hemp cultivar yields a CBD total content of 7.68% and a THC total content of 0.25%. The female plants of CW were cultivated in a greenhouse under controlled conditions, with a light/dark cycle of 18/6 h at the temperature of 25/21 °C (day/night) and 70% humidity. After 8 weeks of vegetative growth, the flowering phase was induced by altering the light/dark regime to 12/12 h.

### Analytical standards

Thirteen cannabinoid standard solutions, cannabinchromene (CBC, 98.8%), cannabichromenic acid (CBCA, 99.8%), cannabidiol (CBD, 99.6%), cannabidiolic acid (CBDA, 99.3%), cannabigerol (CBG, 99.2%), cannabigerolic acid (CBGA, 99.3%) cannabinol (CBN, 99.1%), cannabidivarin (CBDV, 99.2%), cannabidivarinic acid (CBDVA, 99.3%), Δ^9^-tetrahydrocannabinol (Δ^9^-THC, 99.3%), tetrahydrocannabinolic acid (THCA, 99.1%), Δ^9^-tetrahydrocannabivarin (Δ^9^-THCV, 99.1%), and Δ^9^-tetrahydrocannabivarinic acid (Δ^9^-THCVA, 97.9%), at a concentration of 1.0 mg mL^−1^ in methanol or acetonitrile were purchased from Cerilliant (San Antonio, TX, USA). All standards used for this study were the certified reference materials (CRMs).

### Sample preparation of secretory cavity contents by microcapillaries

Pistillate calyx containing numerous capitate-stalked glandular trichomes of female plants in the 8th week of the flowering stage were employed for experiments. The contents of the secretory cavity were acquired using a glass microcapillary (inner diameter 10 μm, MP-010, Micro Support, Japan) under a dissecting microscope (SZ Trinocular, Olympus, Japan). The microcapillary glass tip was utilized to remove the secretory cavity contents from the head parts of glandular trichomes. Due to the highly integrated nature of the secretory cavity, its contents rapidly entered the glass microcapillary the moment the microcapillary tip penetrated the secretory cavity (Fig. 1). Each secretory cavity sample was obtained from 40 glandular trichomes. It was transferred in a vial with a 250 µL insert (Verex, Phenomenex, USA) filled with 150 µL methanol, then promptly moved into a 300 µL Verex amber (Phenomenex, USA) and stored at 4 °C.

### Sample preparation for inflorescence

The inflorescences of female plants in the 8th week of the flowering stage were harvested and air-dried in darkness for 15 days at room temperature. The whole *Cannabis* plant usually takes 14 days to obtain 11% w/w moisture content, which is the most appropriate contents of water to proceed for the cannabinoid analysis^53^. The dried inflorescence was manually ground with a mortar and pestle, and sieved through a mesh (pore size, 1.18 mm). After grinding, 0.1 g of inflorescence powder was transferred to a 20 ml glass scintillation vial, and 20 ml of ethanol (HPLC grade 200-proof) was added. These extracts were placed in a sonication bath (Branson 3510, Branson Ultrasonics Corp, Brookfield, USA) for 30 min at room temperature. Subsequently, the samples were left undisturbed overnight for 18 h in a dark room. The following day, the samples were vortexed and allowed to stand for 10 min. The supernatants were then filtered through a nylon membrane filter (0.45 μm) into a sample vial for high-pressure liquid chromatography (HPLC) analysis.

### HPLC conditions for analysis

The extract samples were subjected to HPLC analysis (Thermo Scientific UltiMate 3000, Waltham, USA). The HPLC was controlled through Chromeleon 7.2 Software, version 7.2 SR5 (Thermo Fisher Scientific, Waltham, USA). The instrument conditions consisted of a column, Accucore aQ C18 Polar Endcapped, 2.1 × 100 mm, 2.6 μm. A sample injection of 2.0 μL, and a flow rate of 0.45 mL/min was utilised. The mobile phase (A) consisted of 0.10% formic acid water and the mobile phase (B) comprised of 0.10% formic acid in methanol B. Separation was performed using a gradient of 62% B initially, increasing to 66% B at 13.75 min, followed by an increase to 80% B at 20 min. This was maintained for 4 min before returning to 62% B for 3.0 min of equilibration between injections. The column temperature was set to 50 °C. Four-point standard curves (5, 10, 50, 100 mg/l) were developed for the cannabinoids using 228 nm from the diode array detector (multiple wavelength detection DAD 3000) to detect the presence of the analyte. The above-mentioned software determined the peak integrations for each of the compounds. We followed the procedures of our previous protocol for the limit of detection and quantification^54^. Posterior analyses were performed to determine the mg/L of acidic and neutral cannabinoids, and finally to identify the content in mg/L in 1 g of CW.

### Data processing

Data processing was performed using GraphPad Prism 10® (GraphPad Software, In., La Jolla, CA). Statistical analysis of means between groups was performed by two-way ANOVA at a 5% significance level (*p* < 0.05). The statistical method was chosen to evaluate the main effects and interaction between two independent variables on our outcome.

## Conclusions

Our findings are the quantitative results that reveal the decarboxylation rate of acidic cannabinoids to neutral cannabinoids throughout the 15-day storage period. Additionally, we quantified the cannabinoids in the secretory cavity content of each trichome. Four acidic cannabinoids (CBDA, THCA, CBCA, and CBGA), four neutral cannabinoids (CBD, Δ^9^-THC, CBC, and CBG), and three degraded cannabinoids (CBDVA, Δ^9^-THCVA, and Δ^9^-THCV) were detected on the inflorescence sample. In contrast, four acidic cannabinoids (CBDA, THCA, CBCA, and CBGA), one neutral cannabinoid (CBD), and one degraded cannabinoid (CBDVA) were detected in the secretory cavity contents. The most notable differences in the content of cannabinoids were the presence of Δ^9^-THCVA and Δ^9^-THCV in the inflorescence sample, while secretory cavity substances did not exhibit these compounds. Instead, CBDVA was exclusively detected in the secretory cavity contents.

In this research, the CBDA content remarkably decreased during the storage period. The ratio of CBD to CBDA was 1:99 (w/w) in secretory cavity contents and 1:20 (w/w) in the inflorescence sample. The contents of CBD increased by five times during the 15-days storage. Based on these results, we can predict the conversion rate of acidic cannabinoids and use these data to infer the shelf-life of hemp products.

## Data Availability

Data is available upon reasonable request.
